# Metagenome Analysis of a Hydrocarbon-Degrading Bacterial Consortium Reveals the Specific Roles of BTEX Biodegraders

**DOI:** 10.3390/genes12010098

**Published:** 2021-01-14

**Authors:** Michael O. Eze

**Affiliations:** 1 Department of Genomic and Applied Microbiology and Göttingen Genomics Laboratory, Georg-August University of Göttingen, 37077 Göttingen, Germany; meze@gwdg.de; 2 Department of Earth and Environmental Sciences and MQ Marine Research Centre, Macquarie University, Sydney, NSW 2109, Australia

**Keywords:** petroleum hydrocarbons, microbial consortium, biodegradation, BTEX activation

## Abstract

Environmental contamination by petroleum hydrocarbons is of concern due to the carcinogenicity and neurotoxicity of these compounds. Successful bioremediation of organic contaminants requires bacterial populations with degradative capacity for these contaminants. Through successive enrichment of microorganisms from a petroleum-contaminated soil using diesel fuel as the sole carbon and energy source, we successfully isolated a bacterial consortium that can degrade diesel fuel hydrocarbons. Metagenome analysis revealed the specific roles of different microbial populations involved in the degradation of benzene, toluene, ethylbenzene and xylene (BTEX), and the metabolic pathways involved in these reactions. One hundred and five putative coding DNA sequences were identified as responsible for both the activation of BTEX and central metabolism (ring-cleavage) of catechol and alkylcatechols during BTEX degradation. The majority of the Coding DNA sequences (CDSs) were affiliated to *Acidocella*, which was also the dominant bacterial genus in the consortium. The inoculation of diesel fuel contaminated soils with the consortium resulted in approximately 70% hydrocarbon biodegradation, indicating the potential of the consortium for environmental remediation of petroleum hydrocarbons.

## 1. Introduction

Industrialization and increasing demand for energy have led to continuous exploitation of fossil fuels. This has resulted in anthropogenic contamination of many aquatic and terrestrial ecosystems. For decades, petroleum hydrocarbons including, but not limited to, benzene, toluene, ethylbenzene and xylene (BTEX) have been of major concern owing to their toxicity to human and animal lives [1,2,3,4,5]. The International Agency for Research on Cancer (IARC) has classified benzene and ethylbenzene as Group 1 and 2B carcinogens, respectively [6]. A number of incidents involving respiratory cancers have been reported among factory workers where BTEX and chlorinated derivatives were produced [7,8].

The stability of aromatic hydrocarbons is often responsible for their resistance to biodegradation. This results in their bioaccumulation and biomagnification along trophic levels of organisms. The degradation requires initial activation by oxygenases. In aerobic degradation, oxygen is both the terminal electron acceptor and a necessary reactant for activating hydrocarbons by converting them into oxygenated intermediates [9]. This process is orchestrated by monooxygenases and dioxygenases that incorporate oxygen atoms, forming alcohols. Central metabolism of aromatic hydrocarbons involves ortho- and meta-cleavage of catechol and alkylcatechols [9,10]. Further oxidation results in the formation of oxoadipate and aldehydes, with the former being metabolised via succinyl-CoA and the latter via acetyl-CoA and propanoyl-CoA [11,12].

Bioremediation of hydrocarbons such as BTEX relies primarily on biodegradation by microorganisms through a series of processes that depend on the nature and amount of the hydrocarbons present [13,14,15]. Bioremediation processes are both environmentally friendly and cost-effective. Correspondingly, the development of microbial consortia for the degradation of BTEX and other petroleum-derived products has attracted increasing attention [16,17,18,19,20], especially the remediation of contaminated wastewaters [21]. The effectiveness of any bioremediation technique depends, inter-alia, on the ability of associated microbes to perform the complex reactions involved in hydrocarbon degradation [11,22,23].

Microorganisms present in contaminated environments often manifest the environmental adaptability required for effective remediation of contaminants [24,25,26]. Hence, petroleum-contaminated sites are often the first point of recourse in the search for organisms with the ability for organic pollutant degradation. This enables the cultivation of microbial consortia that can be used as inocula for environmental remediation of hydrocarbon-contaminated sites [19,27]. The purpose of this study was to isolate a hydrocarbon-degrading consortium and consequently examine the potentials of specific microbial species for BTEX activation and metabolism. The results of this study will expand the range of applicable microorganisms for environmental remediation of BTEX and other organic pollutants.

## 2. Materials and Methods

### 2.1. Soil Sampling and Study Site Description

Three topsoil samples (10 g each) at approximately 5 cm depth were taken around a crude oil seepage point in a heavily polluted location at the historical oil field in Wietze (52°39′0″ N, 09°50′0″ E), Germany in November 2019. The pH of the soil was 6.5. The site is contaminated with crude oil from underground seepages, and organic debris from surrounding plants. The samples were transported to the laboratory on ice and stored at −20 °C until further analysis. Wietze is an important historical site, where industrial extraction of petroleum in Germany began in 1859 [28]. Between 1900 and 1920, Wietze was the most productive German oil field and provided almost 80% of the German production. Although no oil production is currently taking place in the oil field, it still contains sites with heavy crude oil pollution resulting from seepages. It is therefore an ideal source of potential BTEX-degrading microorganism.

### 2.2. Enrichment Culture and Growth Conditions

In order to isolate hydrocarbon-degrading bacteria, the three soil samples were manually mixed together, after which 1 g of soil was added to 100 mL of liquid mineral medium (MM). Mineral medium comprised KH_2_PO_4_ (0.5 g/L), NaCl (0.5 g/L), NH_4_Cl (0.5 g/L). Sterile-filtered trace element solution, vitamin solution and MgSO_4_·7H_2_O (100 mg/mL) were added at 1 mL/L, 1 mL/L, and 5 mL/L post-autoclaving. One ml of sterile-filtered diesel fuel was added as the sole carbon and energy source. The culture was grown at 30 °C with shaking (110 rpm) and maintained within a 5-day subculture. After three subcultures, 30 mL aliquot was centrifuged for 10 min at 4000× *g*. The cell pellets were resuspended in 1.0 mL of MM and mixed with 1.0 mL of glycerol (50%) and deep-frozen at −80 °C. Another 30 mL aliquot was centrifuged for the purpose of metagenome studies. The remaining culture was concentrated at OD_600_ = 1.800 for the purpose of inoculation during the greenhouse experiment.

### 2.3. DNA Extraction

Microbial cells (OD_600_ = 0.635) from 30 mL of the enrichment culture were harvested by centrifugation at 4000× *g* for 10 min. The supernatant was subsequently discarded. DNA from the cell pellets were extracted using the PowerSoil^®^ DNA Extraction kit (Qiagen, Hilden, Germany).

### 2.4. Sequencing of Bacterial 16S rRNA Gene

Bacterial 16S rRNA genes were amplified using the forward primer S-D-Bact-0341-b-S-17 (5′-CCT ACG GGN GGC WGC AG-3′) [29] and the reverse primer S-D-Bact-0785-a-A-21 (5′-GAC TAC HVG GGT ATC TAA TCC-3′) [29] containing Illumina Nextera adapters for sequencing. The PCR reaction (25 µL) contained 5 µL of five-fold Phusion HF buffer, 200 µM of each of the four deoxynucleoside triphosphates, 4 µM of each primer, 1 U of Phusion high fidelity DNA polymerase (Thermo Scientific, Waltham, MA, USA), and approximately 50 ng of the extracted DNA as a template. The negative control was performed by using the reaction mixture without a template. The following thermal cycling scheme was used: initial denaturation at 98 °C for 30 s, 30 cycles of denaturation at 98 °C for 15 s, annealing at 53 °C for 30 s, followed by extension at 72 °C for 30 s. The final extension was carried out at 72 °C for 2 min. The obtained PCR product was controlled for appropriate size and purified using the MagSi-NGS Plus kit according to the manufacturer’s protocol (Steinbrenner Laborsysteme GmbH, Germany). The quantification of the PCR product was performed using the Quant-iT dsDNA HS assay kit and a Qubit fluorometer, as recommended by the manufacturer (Thermo Scientific). The DNA sample was barcoded using the Nextera XT-Index kit (Illumina, San Diego, CA, USA) and the Kapa HIFI Hot Start polymerase (Kapa Biosystems, Wilmington, MA, USA). Sequencing was performed at the Göttingen Genomics Laboratory on an Illumina MiSeq Sequencing platform (paired end 2 × 300 bp) using the MiSeq Reagent kit v3, as recommended by the manufacturer (Illumina).

### 2.5. Processing of the 16S rRNA Gene Data

Trimmomatic version 0.39 [30] was initially used to truncate low quality reads if quality dropped below 12 in a sliding window of 4 bp. The dataset was subsequently processed with Usearch version 11.0.667 [31] as described in F. Wemheuer et al. [32]. In brief, paired end reads were merged and quality-filtered. Filtering included the removal of low-quality reads (maximum number of expected errors >2 and more than 1 ambitious base, respectively) and those shorter than 200 bp. Processed sequences were joined, dereplicated and clustered in zero-radius operational taxonomic units (zOTUs) using the UNOISE algorithm implemented in Usearch. A de novo chimera removal was included in the clustering step. Afterwards, zOTU sequences were taxonomically classified using the SINTAX algorithm against the SILVA database (SILVA SSURef 138 NR99). All non-bacterial zOTUs were removed based on their taxonomic classification. Subsequently, processed sequences were mapped on final zOTU sequences to calculate the distribution and abundance of each OTU in the consortium.

### 2.6. Metagenome Sequencing, Assembly and Analysis

Paired-end sequencing libraries were generated from environmental DNA and barcoded using the Nextera XT-Index kit (Illumina, San Diego, CA, USA) and the Kapa HIFI Hot Start polymerase (Kapa Biosystems, Wilmington, MA, USA). The Göttingen Genomics Laboratory determined the sequences employing an Illumina HiSeq 2500 system using the HiSeq Rapid SBS kit V2 (2 × 250 bp) as recommended by the manufacturer (Illumina). Metagenomic reads were further processed as described previously [24]. In brief, reads were processed with the Trimmomatic tool version 0.39 [30] and assembled using metaSPAdes version 3.13.2 [33]. Coverage information for each scaffold was determined using Bowtie2 version 2.3.2 [34] and SAMtools version 1.7 [35].

### 2.7. Identification of CDSs Involved in BTEX Biodegradation

Coding DNA sequences (CDSs) of putative enzymes involved in the degradation of BTEX were identified in the bacterial metagenome by annotations with prodigal version 2.6.3 [36]. Functional annotation was performed with diamond version v0.9.29 [37] and the KEGG database (October release 2018) [38]. The enzymes of interest include benzene, toluene, xylene, and ethylbenzene monooxygenases and dioxygenases, toluene methyl monooxygenase, phenol 2-monooxygenase, benzoate/toluate 1,2-dioxygenase, benzaldehyde dehydrogenase, aryl-alcohol dehydrogenase, dihydroxycyclohexadiene carboxylate dehydrogenase, catechol 1,2-dioxygenase, catechol 2,3-dioxygenase, muconate cycloisomerase, muconolactone D-isomerase, 3-oxoadipate enol-lactonase, 2-hydroxymuconate-semialdehyde hydrolase, 2-hydroxymuconate-6-semialdehyde dehydrogenase, 4-oxalocrotonate tautomerase, 2-oxo-3-hexenedioate decarboxylase, 2-keto-4-pentenoate hydratase, 4-hydroxy 2-oxovalerate aldolase, acetaldehyde dehydrogenase, 3-methylcatechol 2,3-dioxygenase, 2,3-dihydroxyethylbenzene 1,2-dioxygenase, and 2-hydroxy-6-oxo-octa-2,4-dienoate hydrolase.

### 2.8. Greenhouse Biodegradation Experiment

The soil used for this experiment was “Primaster turf.” Primaster turf is made from a mixture of unsterilized screened sand, soil, and composted organics. The soil textural class was determined as sand (88.6% sand, 6.1% silt and 5.3% clay) with 12.5% organic matter content by loss on ignition. The soil had a total nitrogen content of 0.15% and a pH of 7.1. The soil was initially homogenized by sieving through a 2-mm sieve to remove unwanted large particles. Diesel fuel contaminated soils (5 g/kg) were prepared following the methods of M. O. Eze et al. [39]; 30 mL of the consortium was centrifuged at 4000× *g* for 10 min. The microbial cells were washed twice in mineral medium and concentrated at OD_600_ = 1.800 to be used as microbial inocula. The cells were inoculated to the pots containing 5 g/kg diesel fuel contaminated soils. Uninoculated contaminated pots were set up as controls. The whole experiment was performed in triplicates in a greenhouse, and pots were watered once a week for a period of 60 days. After 60 days, the soils were collected for geochemical analysis.

### 2.9. Geochemical Analysis of Biodegradation

#### 2.9.1. Extraction of Residual Hydrocarbons

After 60 days, the soil samples per pot were thoroughly homogenized as described in M. O. Eze et al. [39]. For hydrocarbon analyses, 1 g of the ground freeze-dried soils from each pot was further homogenized with a small amount of sodium sulfate (Na_2_SO_4_) and transferred into a Teflon microwave digestion vessel. The samples were extracted two times with 2.5 mL n-hexane each in a microwave device (Mars Xpress, CEM; 1600W, 100 °C, 20 min). For reference, 2.5 µL diesel fuel (density = 0.82 g/mL) were dissolved in 5 mL n-hexane instead of 1 g soil sample. The extracts were combined into 7 mL vials and topped to 5 mL with n-hexane. A 20% aliquot (1 mL) of each extract was pipetted into a 2 mL autosampler vial, and 20 µL n-icosane D42 (200 mg/L) was added as an internal quantification standard.

#### 2.9.2. Molecular Analysis of Biodegradation

Gas chromatography-mass spectrometry (GC-MS) analyses of the samples were performed using a Thermo Scientific Trace 1300 Series GC coupled to a Thermo Scientific Quantum XLS Ultra MS. The GC capillary column used was a Phenomenex Zebron ZB–5MS (30 m, 0.1 µm film thickness, inner diameter 0.25 mm). Compounds were transferred splitless to the GC column at an injector temperature of 300 °C. Helium was used as the carrier gas at a flow rate of 1.5 mL/min. The GC temperature program was as follows: 80 °C (hold 1 min), 80 °C to 310 °C at 5 °C/min (hold 20 min). Electron ionization mass spectra were recorded at 70 eV electron energy in full scan mode (mass range *m/z* 50–600, scan time 0.42 s). Peak areas were integrated using Thermo Xcalibur software version 2.2 (Thermo Fisher Scientific Inc., Waltham, MA, USA).

### 2.10. Statistical Analysis

All statistical analysis were performed using R language [40]. To determine if remediation efficiencies of the inoculated treatments and the uninoculated controls (representing natural attenuation) were significantly different from each other and also different from the nominal soil diesel fuel concentrations, a one-way analysis of variance (ANOVA) was employed, followed by Tukey’s all-pairwise comparisons [41,42]. The Tukey method tests all possible pair of samples, and uses pairwise post-hoc testing to determine whether there is a difference between the means [42]. The *p* values were adjusted using the Bonferroni method. This approach is considered the simplest and most conservative method to control family-wise error rate in multiple comparisons.

## 3. Results

### 3.1. Bacterial Diversity in the Consortium

The 16S rRNA gene sequencing resulted in 18,575 bacterial sequences belonging to 225 operational taxonomic units (zOTUs). The successive enrichments of the polluted soil sample resulted in a consortium that is dominated by *Alphaproteobacteria* (87%) and *Acidobacteriae* (13%) (Figure S1). At the level of genera, *Acidocella* is the most dominant bacterial genus (Figure S2). Other represented relevant genera include *Acidiphilium*, *Acidobacterium*, and *Aquabacter* (Figures S2–S4).

### 3.2. Activation of BTEX

#### 3.2.1. Benzene and Ethylbenzene

**Figure 1:**
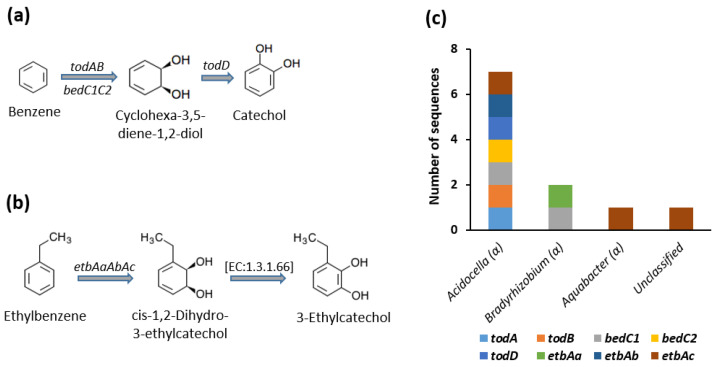
The activation of (**a**) Benzene and (**b**) Ethylbenzene by respective dioxygenases. (**c**) Genus assignation of the genes involved in benzene and ethylbenzene activation. Symbol in parenthesis indicate the taxonomic class of each genus. *todAB* and *bedC1C2*: benzene/toluene dioxygenases; *todD*: cis-1,2-dihydrobenzene-1,2-diol/chlorobenzene dihydrodiol dehydrogenase; *etbAaAbAc*: ethylbenzene dioxygenases.

The results of our metagenome analysis revealed the presence of six putative CDSs that are responsible for the two-step reactions involved in the conversion of benzene to catechol. These include five CDSs classified as benzene dioxygenases and one CDS classified as cis-1,2-dihydrobenzene-1,2-diol/chlorobenzene dihydrodiol dehydrogenase (*todD*) (Figure 1). Taxonomic assignment revealed that five of these CDSs were affiliated to the bacterial genus *Acidocella* (Figure 1c). Ethylbenzene is activated by ethylbenzene dioxygenase with subsequent reduction to 3-ethylcatechol in the cis-1,2-dihydro-3-ethylcatechol oxidoreductase pathway (EC:1.3.1.66). Four of the CDSs involved in these reactions were assigned to *Acidocella* (2 CDSs), *Bradyrhizobium* (1 CDS), and *Aquabacter* (1 CDS).

#### 3.2.2. Toluene and Xylenes

**Figure 2:**
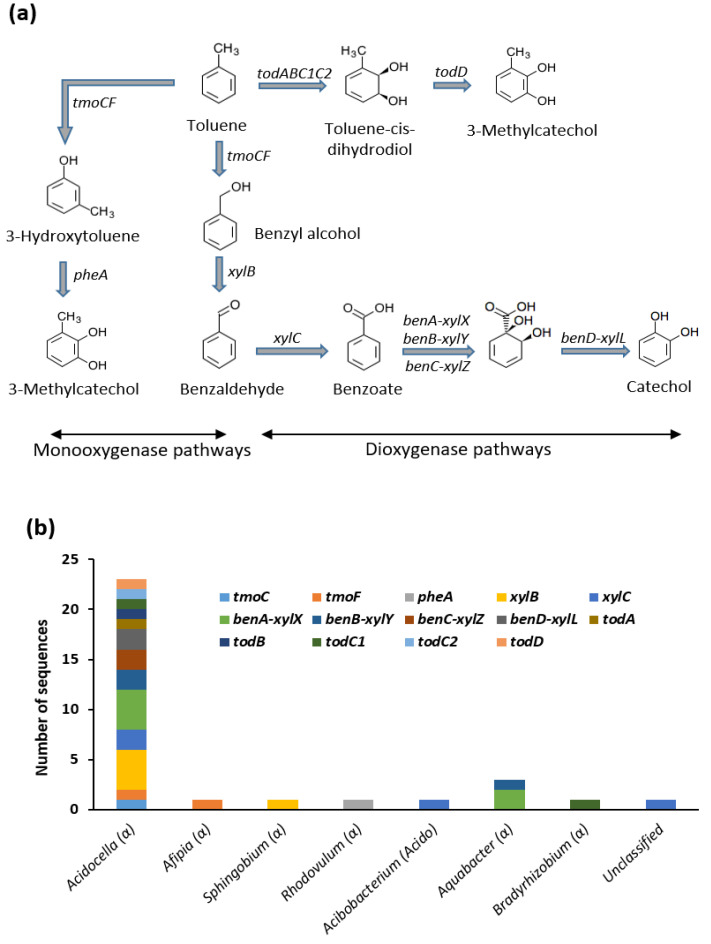
(**a**) Activation of toluene by both monooxygenases and dioxygenases. (**b**) Genus level assignment of the genes involved in toluene activation. Symbol (or letters) in parenthesis indicate the taxonomic class of each genus. *tmoCF*: toluene monooxygenases; *pheA*: phenol 2-monooxygenase; *xylB*: aryl alcohol dehydrogenase; *xylC*: benzaldehyde dehydrogenase; *benA-xylX*, *benB-xylY* and *benC-xylZ*: benzoate/toluate 1,2-dioxygenases; *benD-xylL*: dihydroxycyclohexadiene carboxylate dehydrogenase; *todABC1C2*: benzene/toluene dioxygenases; *todD*: cis-1,2-dihydrobenzene-1,2-diol/chlorobenzene dihydrodiol dehydrogenase.

Our results revealed that our consortium have the genes involved in all oxidation steps for the conversion of toluene to either catechol or 3-methylcatechol. These include the *tmoCF*, *pheA*, and *xylB* genes that are involved in the monooxygenase pathway, as well as the *todABC1C2D*, *xylC*, *benA-xylX*, *benB-xylY*, *benC-xylZ*, and *benD-xylL* genes involved in the dioxygenase pathway (Figure 2). The degradation of ortho-, meta-, and para-xylenes are initiated by toluene methyl monooxygenase genes *xylM* and *xylA*. However, benzaldehyde dehydrogenase (*xylC*), benzoate/toluate 1,2-dioxygenases (*benA-xylX*, *benB-xylY* and *benC-xylZ*), and dihydroxycyclohexadiene carboxylate dehydrogenase (*benD-xylL*) were involved in subsequent oxidation of 2-methylbenzaldehyde, 3-methylbenzaldehyde and 4-methylbenzaldehyde to 3-methylcatechol and 4-methylcatechol. A total of 32 CDSs were found in our metagenome data that are responsible for the activation of toluene and xylenes. Genus level assignments indicate that the majority of these CDSs (8 CDSs) belong to *Acidocella* (Figure 2b).

### 3.3. Central Metabolism of BTEX

#### 3.3.1. Ortho-Cleavage of Catechol

**Figure 3:**
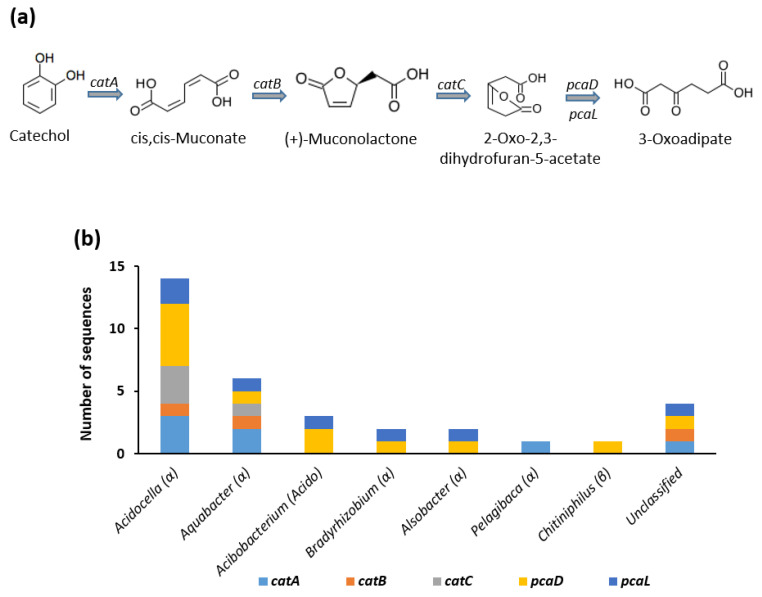
(**a**) Ortho-cleavage of catechol. (**b**) Genus assignation of the genes involved in ortho-cleavage of catechol and subsequent degradation reactions. Symbol (or letters) in parenthesis indicate the taxonomic class of each genus. *catA*: catechol 1,2-dioxygenase; *catB*: muconate cycloisomerase; *catC*: muconolactone D-isomerase; *pcaDL*: 3-oxoadipate enol-lactonases.

The results of our study revealed that our bacterial consortium contains the CDSs required for ortho-cleavage of catechol. These include catechol 1,2-dioxygenase (*catA*), muconate cycloisomerase (*catB*), muconolactone D-isomerase (*catC*), and 3-oxoadipate enol-lactonases (*pcaD* and *pcaL*) (Figure 3). Of the 33 coding DNA sequences involved in this pathway, 14 belong to *Acidocella* and 6 to *Aquabacter*. Other represented genera include *Acidobacterium* (3 CDSs), *Bradyrhizobium* (2 CDSs), *Alsobacter* (2 CDSs), *Pelagibaca* (1 CDS), and *Chitiniphilus* (1 CDS). Four of the CDSs could not be assigned to any specific bacterial genus (Figure 3b).

#### 3.3.2. Meta-Cleavage of Catechol, Methylcatechol, and Ethylcatechol

**Figure 4:**
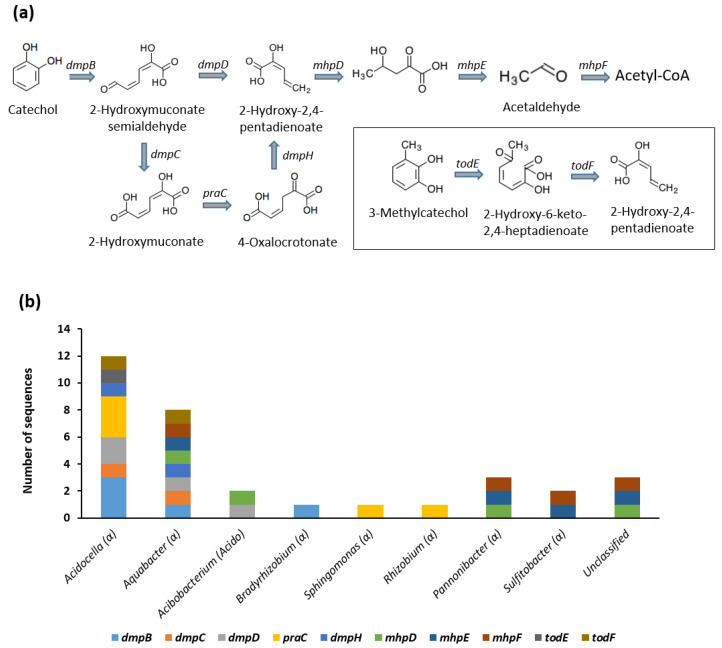
(**a**) Meta-cleavage of catechol and 3-methylcatechol. (**b**) Genus level assignment of the genes involved in meta-cleavage of catechol and 3-methylcatechol and their subsequent degradation reactions. Symbol (or letters) in parenthesis indicate the taxonomic class of each genus. *dmpB*: catechol 2,3-dioxygenase; *dmpC*: 2-hydroxymuconate-6-semialdehyde dehydrogenase; *dmpD*: 2-hydroxymuconate-semialdehyde hydrolase; *praC*: 4-oxalocrotonate tautomerase; *dmpH*: 2-oxo-3-hexenedioate decarboxylase; *mhpD*: 2-keto-4-pentenoate hydratase; *mhpE*: 4-hydroxy 2-oxovalerate aldolase; *mhpF*: acetaldehyde dehydrogenase; *todE*: 3-methylcatechol 2,3-dioxygenase; *todF*: 2-hydroxy-6-oxohepta-2,4-dienoate hydrolase.

The degradation of toluene and xylenes produces catechol, 3-methylcatechol, and 4-methylcatechol intermediates. Our results showed that our bacterial consortium is able to degrade catechol and 3-methylcatechol through a series of ring-cleavage and ring-hydroxylating reactions. The CDSs in our consortium that are responsible for these reactions include the *dmpBCDH, praC, mhpDEF,* and *todEF* genes (Figure 4) with a total of 33 CDSs. On the other hand, there is no evidence, from our metagenome data, of the presence of *bphHIJ* genes needed for the degradation of 4-methylcatechol. Taxonomic classification indicated that over 60% of the CDSs belong to two genera namely *Acidocella* and *Aquabacter* (Figure 4b). This was followed by *Pannonibacter* (3 CDSs), *Acidobacterium* (2 CDSs), and *Sulfitobacter* (2 CDSs). Three of the CDSs involved in meta-cleavage pathway could not be assigned to any specific bacterial genus.

On the other hand, the activation of ethylbenzene leads to the formation of 3-ethylcatechol. Therefore, central metabolism of 3-ethylbenzene involves the meta-cleavage of 3-ethylcatechol by the enzyme 2,3-dihydroxyethylbenzene 1,2-dioxygenase. This is followed by a decarboxylating dehydrogenase reaction that results in the formation of 2-hydroxy-2,4-pentadienoate. The genes responsible for these reactions are *etbC* and *etbD*. The metagenome data contains only 2 CDSs involved in these reactions. These CDSs are affiliated to *Acidocella* and *Acidobacterium*.

### 3.4. Analysis of Residual Total Petroleum Hydrocarbons

**Figure 5:**
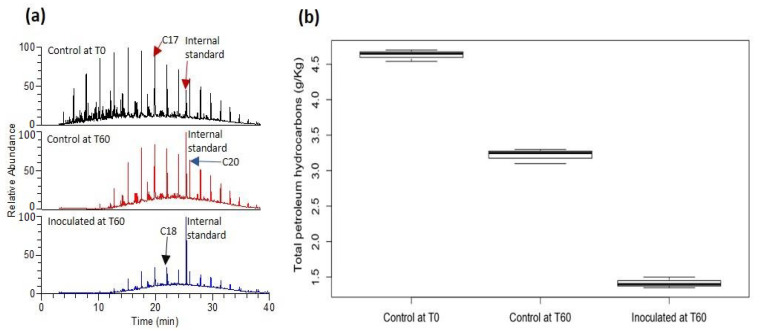
(**a**) Gas chromatography-mass spectrometry (GC-MS) chromatograms (total ion current) showing the relative abundance of nominal diesel fuel hydrocarbons, and residual hydrocarbons after 60 days for control and microbial-inoculated samples. The number associated with each peak represent the number of carbon atoms. (**b**) Boxplot showing nominal total hydrocarbons (Control at T0) and residual total petroleum hydrocarbons after 60 days, under different treatments.

The inoculation of diesel fuel contaminated soils with the isolated consortium resulted in significant degradation of petroleum hydrocarbons (Figure 5). GC-MS analysis showed that the nominal mean concentration (±SE) of diesel fuel in contaminated soil was 4.63 ± 0.05 g/kg. At the end of the 60-day experimental period, natural attenuation reduced the soil diesel fuel concentration to 3.22 ± 0.06 g/kg (Control at T60), while the lowest mean concentration of diesel fuel (1.42 ± 0.04 g/kg) was observed in the inoculated samples (Figure 5). Analysis of variance and pairwise comparison indicated that the mean values were significantly different from each other (Table 1).

## 4. Discussion

Soil contamination with petroleum hydrocarbons and the associated toxicity of these compounds often result in shifts in microbial community composition and reduced microbial diversity by favouring organisms with the ability to utilize organic contaminants as their sole carbon and energy sources. Similarly, successive enrichment of such soil samples with contaminants of interests selectively enriches microbes that can adapt and are able to degrade the target contaminants. The reduction in microbial diversity is due to the toxicity of diesel fuel hydrocarbons, including BTEX, to many microbial communities [43,44]. The toxicity of petroleum hydrocarbons has been linked to its inhibitory effects on the hydrolase activities involved in nitrogen, phosphorus, and carbon cycles [45,46].

Metagenome analysis revealed that the *Acidocella* genus contain most of the genes responsible for the activation of BTEX. The degradation of benzene, toluene, and ethylbenzene begins with their activation by dioxygenases. The metagenome of our consortium contains genes that code for benzene/toluene/chlorobenzene dioxygenase (*todABC1C2*), benzoate/toluate 1,2-dioxygenase (*benA-xylX*, *benB-xylY*, *benC-xylZ*), and ethylbenzene dioxygenase (*etbAaAbAc*) indicating its capacity to activate the degradation of BTEX (Figure 2 and Figure 3). The presence of dioxygenases without such monooxygenases as phenol 2-monooxygenase and toluene methyl-monooxygenase in our metagenome data indicates that our consortium can potentially activate BTEX compounds through the dioxygenase pathway rather than the monooxygenase pathway. The degradation of ortho-, meta-, and para-xylenes follows the same process as that of toluene through benzaldehyde pathway. The only difference is that while toluene is activated by *tmoCF* genes, xylenes are activated by *xylAM* genes and converted to methylbenzyl alcohols [47,48,49]. The *xylAM* genes were not present in our metagenome data. However, the same *xylB*, *xylC*, *benA-xylX*, *benB-xylY*, *benC-xylZ*, and *benD-xylL* genes that were involved in toluene degradation in the consortium were also responsible for the subsequent series of oxidation of methylbenzyl alcohols to their respective methylcatechols (3-methylcatechol and 4-methylcatechol).

Taxonomic assignment revealed that the majority of the CDSs involved in these reactions belong to *Acidocella* (Figure 2c and Figure 3b). This is interesting considering the fact that limited information is available in literature on the potential of *Acidocella* to degrade BTEX compounds [50,51]. Previous studies have often focused on *Pseudomonas* [17,18,19,52,53], and in recent times, some enzymes have been isolated from *Burkholderia* [54,55] and *Paraburkholderia* [20,56,57]. While these genera often contain most of the enzymes involved in aliphatic hydrocarbon degradation and central metabolism of catechol, they sometimes lack a number of enzymes required to completely activate BTEX and polycyclic aromatic hydrocarbons (PAHs). In a study of rhizoremediation of diesel fuel contaminated soils, a scarcity of ring-hydroxylating and ring-cleavage dioxygenases among *Gammaproteobacteria* was reported [27]. In contrast, our results revealed that *Acidocella* genus contain the full range of CDSs involved in the activation of benzene, toluene, and ethylbenzene.

The taxa that are dominant in the enrichment culture were also associated with hydrocarbon enrichment in previous studies of other locations. Similar to our results, previous studies by W. F. M. Röling et al. [50] and C. C. Obieze et al. [51] associated a number of *Alphaproteobacteria*, predominantly *Acidiphilium* and *Acidocella*, with natural oil seepages and acidic conditions. Similarly, *Acidocella facilis,* which is capable of degrading aromatic hydrocarbons, was isolated from a coal runoff basin in South Carolina, USA [58]. Other studies that investigated the remediation of multiple contaminants such as heavy metals and aliphatic hydrocarbons under acidic conditions identified *Acidocella* as a potential genus for the bioremediation of soils and wastewaters contaminated with toxic organic compounds [14,59]. Thus, *Acidocella*’s biodegradative ability and tolerance to heavy metals make them suitable for the remediation of acidic mine sites.

The degradation of BTEX results in the formation of predominantly catechol [9]. Other important intermediates include 3-methylcatechol, 4-methylcatechol, and 3-ethylcatechol. Therefore, central metabolism of BTEX requires ortho-cleavage and/or meta-cleavage of these aromatic rings [11]. Ortho-cleavage of catechol was initiated by catechol 1,2-dioxygenase (*catA*). This was followed by a series of decarboxylating dehydrogenase reactions resulting in the formation of 3-oxoadipate (Figure 3). On the other hand, meta-cleavage of catechol and 3-methylcatechol was carried out by catechol 2,3-dioxygenase (*dmpB*) and 3-methylcatechol 2,3-dioxygenase (*todE*), respectively. A series of hydroxylating and decarboxylating reactions results in the formation of acetaldehyde, which is metabolised by acetyl-CoA (Figure 4). Similarly, *dmpBCD*, *praC*, and *dmpH* genes are responsible for the meta-cleavage of 4-methylcatechol and its degradation to 2-hydroxy-cis-hex-2,4-dienoate [60,61,62]. The degradation of ethylbenzene produces 3-ethylcatechol. As with 3-methylcatechol, the central metabolism of 3-ethylcatechol involves meta-cleavage of the catechol ring. In the case of 3-ethylcatechol, the responsible enzyme is the 2,3-dihydroxyethylbenzene 1,2-dioxygenase (*etbC*) rather than the catechol 2,3-dioxygenase genes (*dmpB* or *catE*). This results in the formation of 2-hydroxy-2,4-pentadienoate and eventual metabolism via acetyl-CoA. The ability of our consortium to metabolize catechol intermediates through the acetyl-CoA pathway indicates its potential biotechnological application for environmental remediation and reclamation of sites contaminated by BTEX compounds and other petroleum hydrocarbons.

Our results revealed that *Acidocella* and *Aquabacter* have the highest potential for the degradation of catechol, 3-methylcatechol, 4-methylcatechol and 3-ethylcatechol (Figure 4b and Figure 5b). The number of CDSs and microbial genera involved in the degradation of catechol and its alkyl substituents were noticeably higher than those involved in the activation reactions. Previous studies have shown that while many microorganisms lack the metabolic ability to activate some aromatic hydrocarbons, they are able to metabolize its intermediates such as catechol and benzoates [11,63,64,65,66,67,68,69]. The activation reactions are often the rate-limiting step in microbial degradation of organic contaminants. The presence of a considerable number of CDSs in the consortium that are needed for both the activation and central metabolism of BTEX indicates the strong potential of the microbial community, and especially *Acidocella* genus for environmental remediation of benzene, toluene, ethylbenzene, and xylenes.

The geochemical analysis of biodegradation revealed that the consortium significantly enhanced the degradation of diesel fuel hydrocarbons (Figure 5). Analysis of variance shows that the amount of residual hydrocarbons in both the inoculated soils and control were significantly different from the nominal concentrations indicating that both microbial inoculation and natural attenuation impacted on hydrocarbon degradation (Table 1). However, while natural attenuation led to only a 30% degradation of petroleum hydrocarbons within the 60-day period, microbial inoculation resulted in approximately 70% degradation of diesel fuel hydrocarbons (Figure 5b). With the majority of the CDSs putatively encoding for monooxygenases and dioxygenases affiliated to the dominant population (*Acidocella*), our results showed that *Acidocella* was responsible for the activation reactions involved in hydrocarbon degradation by the consortium. On the other hand, both *Acidocella* and *Aquabacter* were putatively involved in the central metabolic processes. The low representation of *Aquabacter* in the metagenome data (Supplementary Figure S4) may be attributed to the fact that this genus has only one species—*Aquabacter spiritensis*. In any case, our results revealed that the most effective bacterial genus in the consortium is *Acidocella.* The isolated consortium can have biotechnological application for effective remediation of soils contaminated with BTEX hydrocarbons or other organic pollutants.

## Figures and Tables

**Table 1:** Statistical parameters for the one-way analysis of variance.

Linear Hypothesis (Tukey Contrasts)	Estimate	Std. Error	*t* Value	Pr(>|t|)
Control at T60–Control at T0 == 0	−1.4133	0.0721	−19.61	3.42e-06 ***
Inoculated at T60–Control at T0 == 0	−3.2133	0.0721	−44.59	2.56e-08 ***
Inoculated at T60–Control at T60 == 0	−1.8000	0.0721	−24.98	8.13e-07 ***

Significant codes: 0 ‘***’ 0.001. (Adjusted *p* values—Bonferroni method).

## Data Availability

Raw sequencing data are available at the NCBI Sequence Read Archive (SRA) under accession numbers SRR11310428, SRR11310429, SRR11310430, and SRR11310431.

## Supplementary Materials

The following are available online at https://www.mdpi.com/2073-4425/12/1/98/s1, Figure S1: Taxonomic classification of the bacterial consortium at class level, Figure S2: Taxonomic classification of Alphaproteobacteria at the genus level (“more” refers to additional taxa under the same genus), Figure S3: Taxonomic classification of Acidobacteria at the genus level (“more” refers to additional taxa 20 under the same genus), Figure S4: Taxonomic classification of *Hyphomicrobiaceae* showing *Aquabacter*.
