# Supplementary material for: Metagenome Analysis of a Hydrocarbon-Degrading Bacterial Consortium Reveals the Specific Roles of BTEX Biodegraders
Source: Genes (Basel). 2021 Jan 14;12(1):98. doi: 10.3390/genes12010098 (PMC7828808; doi:10.3390/genes12010098)
Supplement: Supplementary file 1 [file genes-12-00098-s001.pdf]

Metagenome analysis of a hydrocarbon-degrading bacterial consortium reveals the specific roles of BTEX biodegraders

Michael O. Eze[1,2]*

[1]Department of Genomic and Applied Microbiology and Göttingen Genomics Laboratory, Georg-August University of Göttingen, 37077 Göttingen, Germany.

[2]Department of Earth and Environmental Sciences and MQ Marine Research Centre, Macquarie University, Sydney, NSW 2109, Australia.

*Correspondence: meze@gwdg.de

Supplementary Materials

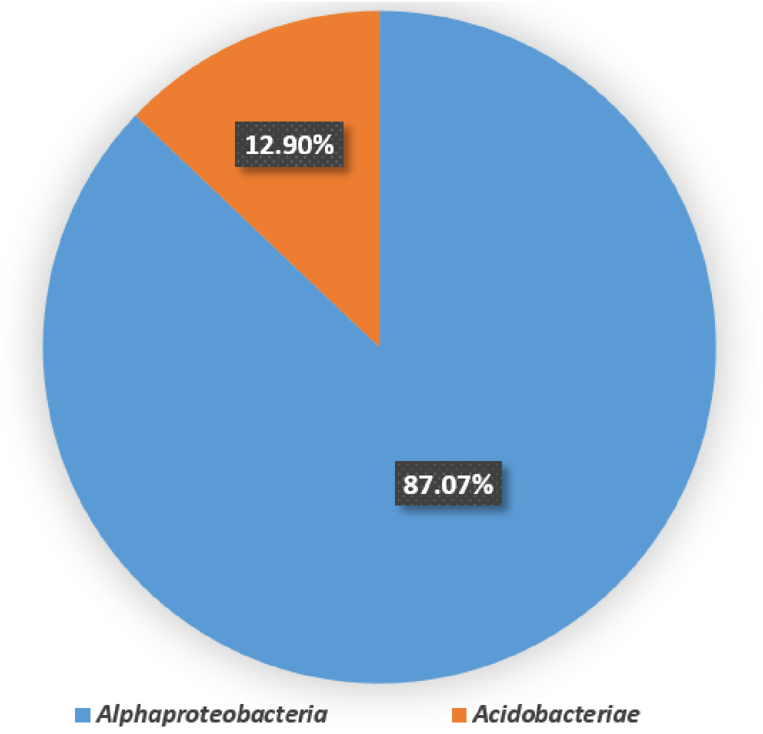

Figure S1. Taxonomic classification of the bacterial consortium at class level.

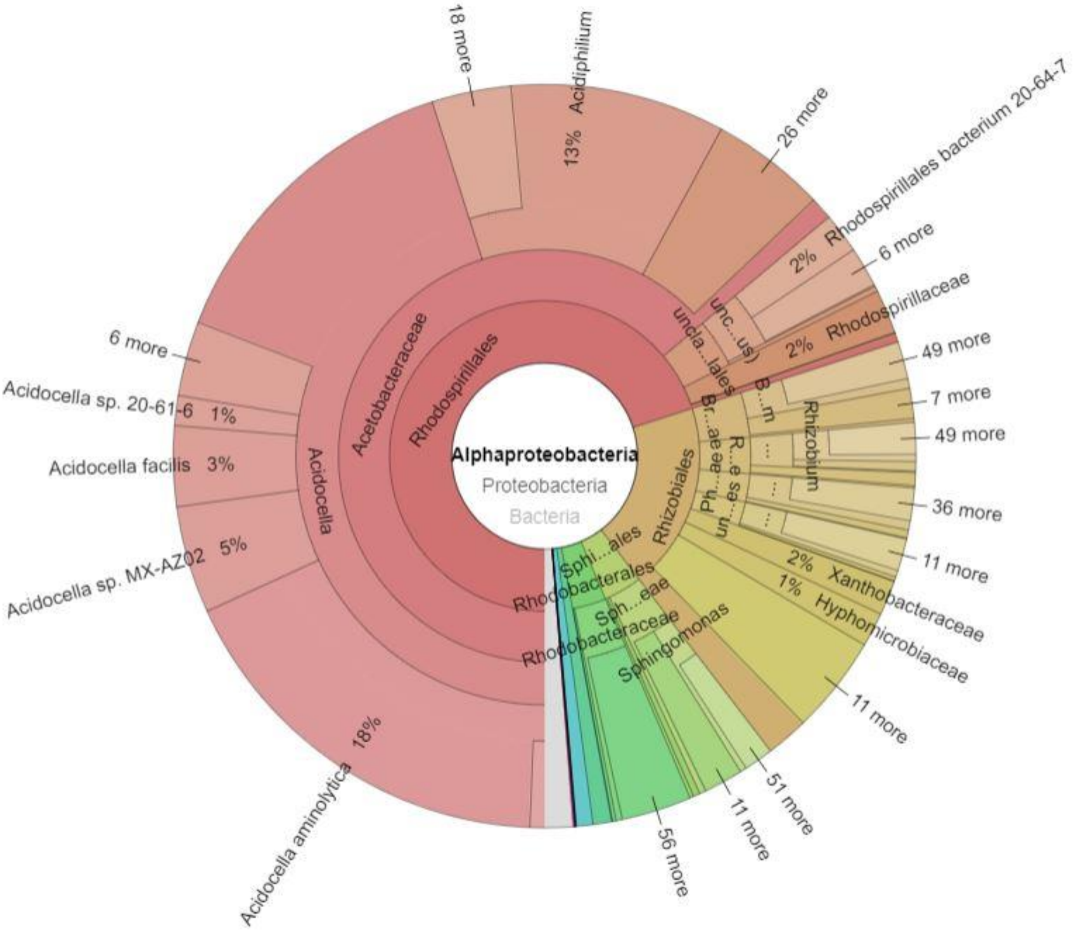

Figure S2. Taxonomic classification of Alphaproteobacteria at the genus level ("more" refers to additional taxa under the same genus).

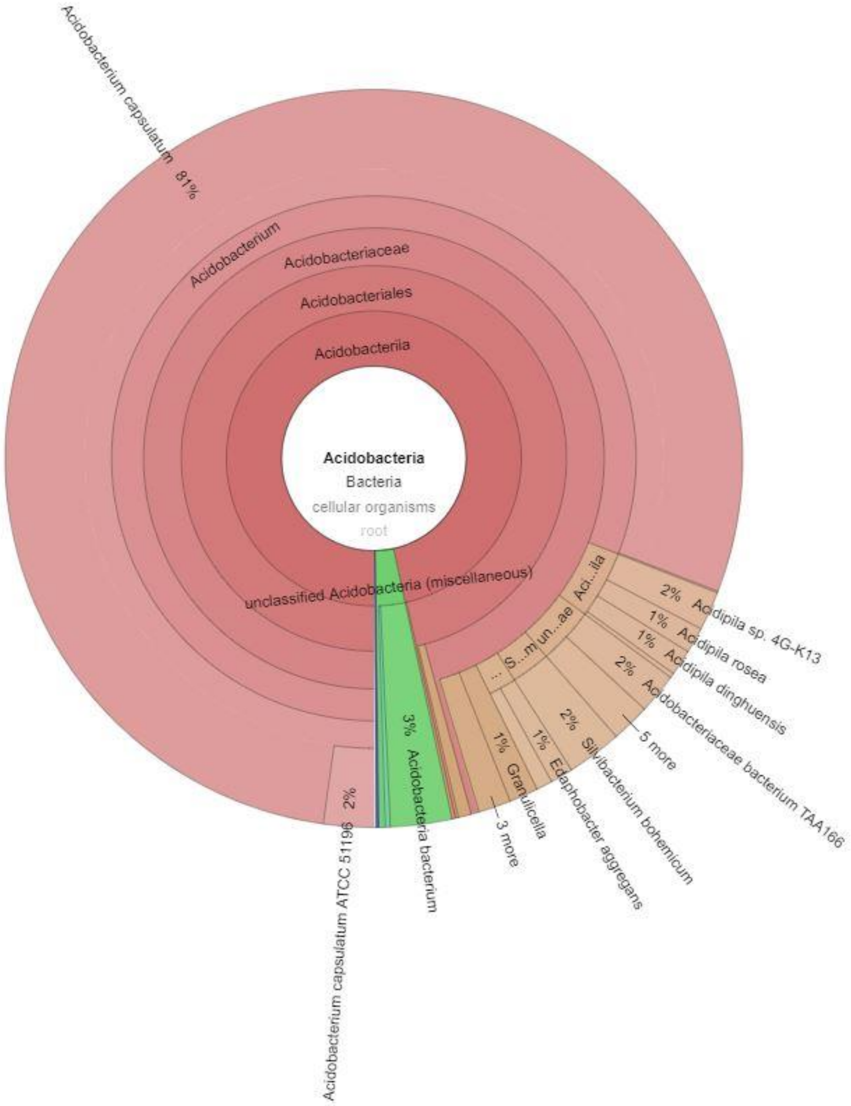

Figure S3. Taxonomic classification of Acidobacteria at the genus level ("more" refers to additional taxa under the same genus).

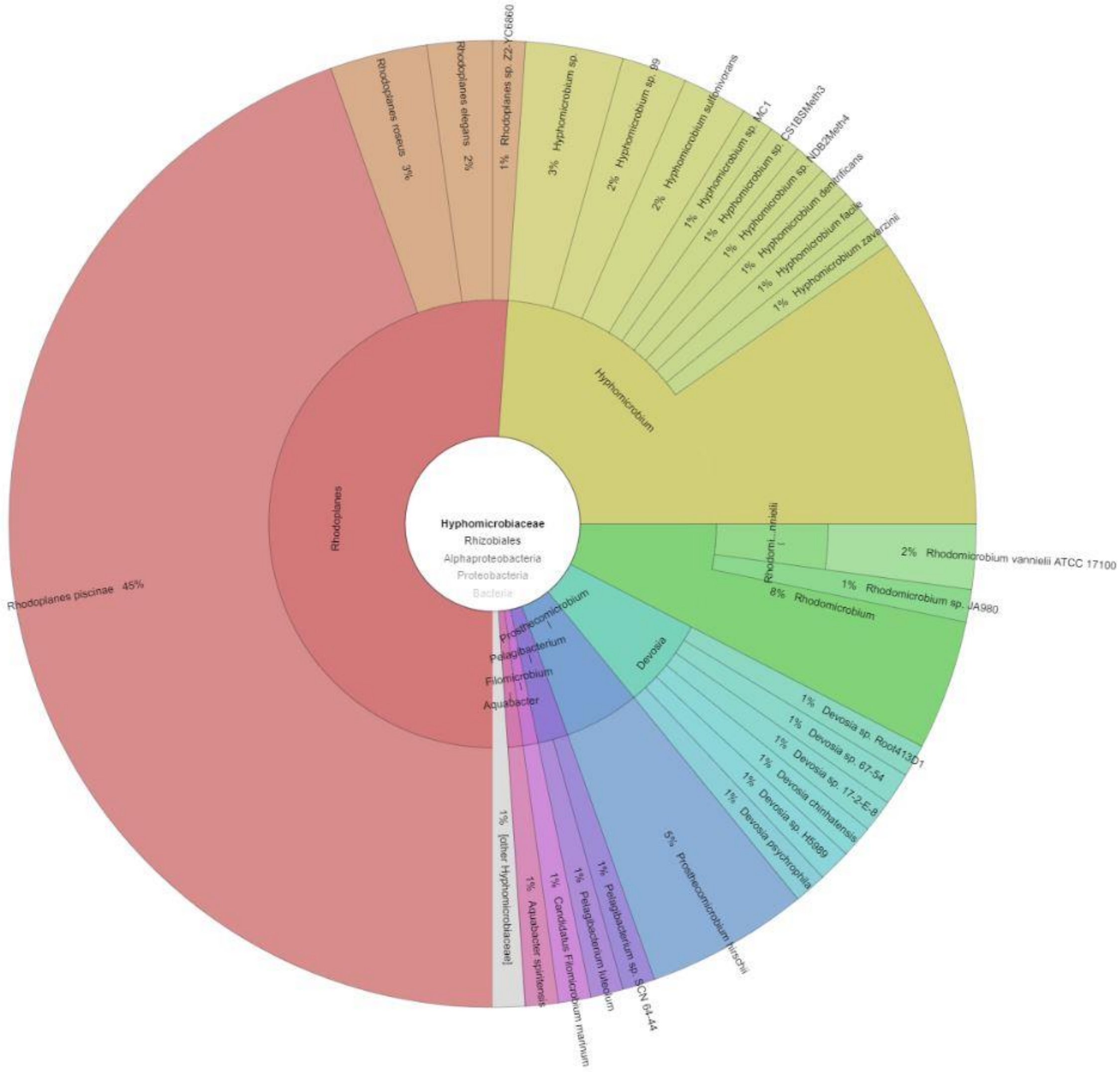

Figure S4. Taxonomic classification of *Hyphomicrobiaceae* showing *Aquabacter*.
